# Identification of Putative Biomarkers for the Early Stage of Porcine Spermatogonial Stem Cells Using Next-Generation Sequencing

**DOI:** 10.1371/journal.pone.0147298

**Published:** 2016-01-22

**Authors:** Won-Young Lee, Jeong Tae Do, Chankyu Park, Jin Hoi Kim, Hak-Jae Chung, Kyung-Woon Kim, Chang-Hyun Gil, Nam-Hyung Kim, Hyuk Song

**Affiliations:** 1 Department of Food Bioscience, Research Institute for Biomedical & Health Science, College of Biomedical & Health Science, Konkuk University, Chung-ju 380–701, Republic of Korea; 2 Department of Animal Biotechnology, Konkuk University, 1 Hwayang-dong, Gwangjin-gu, Seoul 143–701, Republic of Korea; 3 Animal Biotechnology Division, National Institute of Animal Science, RDA, Wanju-gun 565–851, Republic of Korea; 4 School of Medicine, Konkuk University, 1 Hwayang-dong, Gwangjin-gu, Seoul 143–701, Republic of Korea; 5 Department of Animal Science, College of Agriculture, Chungbuk National University, Choung-ju 361–763, Republic of Korea; Qingdao Agricultural University, CHINA

## Abstract

To identify putative biomarkers of porcine spermatogonial stem cells (pSSCs), total RNA sequencing (RNA-seq) analysis was performed on 5- and 180-day-old porcine testes and on pSSC colonies that were established under low temperature culture conditions as reported previously. In total, 10,184 genes were selected using Cufflink software, followed by a logarithm and quantile normalization of the pairwise scatter plot. The correlation rates of pSSCs compared to 5- and 180-day-old testes were 0.869 and 0.529, respectively and that between 5- and 180-day-old testes was 0.580. Hierarchical clustering data revealed that gene expression patterns of pSSCs were similar to 5-day-old testis. By applying a differential expression filter of four fold or greater, 607 genes were identified between pSSCs and 5-day-old testis, and 2118 genes were identified between the 5- and 180-day-old testes. Among these differentially expressed genes, 293 genes were upregulated and 314 genes were downregulated in the 5-day-old testis compared to pSSCs, and 1106 genes were upregulated and 1012 genes were downregulated in the 180-day-old testis compared to the 5-day-old testis. The following genes upregulated in pSSCs compared to 5-day-old testes were selected for additional analysis: matrix metallopeptidase 9 (MMP9), matrix metallopeptidase 1 (MMP1), glutathione peroxidase 1 (GPX1), chemokine receptor 1 (CCR1), insulin-like growth factor binding protein 3 (IGFBP3), CD14, CD209, and Kruppel-like factor 9 (KLF9). Expression levels of these genes were evaluated in pSSCs and in 5- and 180-day-old porcine testes. In addition, immunohistochemistry analysis confirmed their germ cell-specific expression in 5- and 180-day-old testes. These finding may not only be useful in facilitating the enrichment and sorting of porcine spermatogonia, but may also be useful in the study of the early stages of spermatogenic meiosis.

## Introduction

Spermatogenesis in mammals begins at puberty and continues throughout life. The process is initiated and maintained by a small population of spermatogonial stem cells (SSCs), attached to the basement membrane of the seminiferous tubules [1]. The identity of the spermatogonial stem cells has not been fully elucidated, but they have the potential to colonize and restore spermatogenesis in germ cell depleted recipient testes [2, 3].

SSCs exhibit several distinct phenotypes including high expression of α-6 and β1 integrins and lack of expression of the c-kit receptor and α-5 integrin [4–6]. Previous studies have shown that mouse SSCs can be described by their cell-surface phenotype where (CD) 9 and CD90, glial cell derived neurotrophic factor receptor alpha 1 (GFRα1), and cadherin-1 genes are differentially expressed [7–10]. Although, the specific biomarkers of SSCs in non-rodent species have not been fully defined, protein gene product 9.5 (PGP9.5), lectin Dolichos biflorus agglutinin (DBA), promyelocytic leukemia zinc finger (PLZF), Nanog, Oct4, glial cell line-derived neurotrophic factor receptor alpha 1 (GFRα-1), stage-specific embryonic antigen-1 (SSEA-1), undifferentiated embryonic cell transcription factor 1 (UTF1), and SOX2 have been used as markers for porcine spermatogonia [11–19]. However, identification of additional SSC-specific biomarkers is required to understand self-renewal and differentiation of the germ cells.

In the present study, we measured the relative expression level of genes in different stages of testicular development by using sequencing-based tools in *in vitro* cultured porcine SSCs (pSSCs); 5-day-old porcine testicular cells, which are the source of *in vitro* cultured pSSCs; and 180-day-old porcine testicular cells, which contain relatively few spermatogonia and more differentiating cells. We hypothesized that analyzing the relative expression of genes in different stages of testicular development may help identify genes with spermatogonia-specific expression that may be biomarkers of pSSCs. Similar approaches using RNA sequencing-based transcriptome analysis have been used to identify biomarkers in breast [20], colorectal [21], thyroid [22], and metastatic prostate cancer [23]. This high-throughput sequencing technology can identify rare transcripts, isoform genes due to alternative splicing, and differential transcription levels [24]. In addition, the sequencing-based quantitative analysis used in this technology compensates for the lower sensitivity of conventional microarrays in detecting the expression of rare mRNAs and false positives caused by cross-hybridization of highly similar sequences [25, 26].

In this study, we performed high-throughput RNA sequencing to profile the gene expression changes in pSSCs, as well as in 5-day and 180-day-old porcine testes (pTestes). By analyzing these transcriptomes, we identified a number of expressed genes specific to pSSCs and spermatogenesis.

## Materials and Methods

### In-vitro culture of pSSCs

Five-day-old pTestes were obtained from crossbred piglets (Landrace × Large White Yorkshire) from the Sam-Woo breeding farm, Yang Pyoung, Korea. Castration was not conducted as part of this study but was performed in accordance with the farm management plan. pTestes (180-day-old) from Landrace × Large White Yorkshire piglets were donated by the Daesung Corporation (slaughter house), Chung Ju, Korea. The derivation of pSSCs and *in-vitro* culture method was described in our previous study [16]. Briefly, testes were collected from 5-day-old piglets, dissociated with collagenase IV, and meshed using a 40-μm mesh. The red blood cells (RBCs) were removed using RBC lysis buffer. The isolated cells were seed onto 0.2% gelatin-coated cell culture dish and incubated 31°C in 5% CO_2_. Stempro-34 medium was used for pSSC culture with various growth factors.

### RNA extraction and reverse transcription-polymerase chain reaction (RT-PCR)

RNA was extracted from pSSCs and total cells of 5- and 180-day-old pTestes using Qiagen RNA extraction kit according to the manufacturer’s instructions (Qiagen, Venlo, Netherlands). cDNA was synthesized from 1 μg of total RNA using RT-PCR premix kit (iNtRON, Seongnam, South Korea). Target gene PCR amplification was performed with 30 cycles of 30 s at 95°C, 30 s at 55°C, and 30 s at 72°C. All primer sets are listed in S1 Table.

### Real-time PCR

The relative amounts of putative pSSC-specific marker genes mRNAs were estimated in duplicate samples by fluorescence and quantified using Qiagen Rotor gene PCR detection system. The reaction was initiated in a total volume of 20 μL, which contained 10 ng of cDNA and 1 pM of each primer, in a reaction buffer containing iQ SYBR Green Supermix (170–8880; Bio-Rad Laboratories). The cycle threshold (Ct) values were normalized against beta-2-myoglobin (B2M) gene expression. The results were expressed as target gene expression relative to control gene expression. PCR amplification was performed with 40 cycles of 20 s at 95°C, 20 s at 55°C, and 20 s at 72°C with the same primer sets used for real-time PCR.

### RNA isolation and RNA library construction for high-throughput sequencing

For RNA library construction, total RNA was extracted from passage 2–3 of pSSCs, 5-, and 180-day-old porcine testes. Quality and quantity of total RNA were determined using a NanoDrop 1000 spectrophotometer (Thermo Scientific, DE, USA) and Bioanalyzer 2100 (Agilent Technologies, Carlsbad, CA, USA). Illumina-compatible libraries were constructed using a TruSeq RNA Library Preparation Kit (Illumina, San Diego, CA, USA) according to the manufacturer’s instructions. Briefly, messenger RNA was purified by poly A selection of total RNA. Single-stranded cDNAs were synthesized with random hexamer primers by using the chemically fragmented and converted mRNA; then, complementary cDNA strands were synthesized to generate double-stranded (ds) cDNAs for the construction of the TruSeq library. The short ds-cDNA fragments were connected using sequencing adapters, and appropriate fragments were separated by agarose gel electrophoresis. Finally, the RNA libraries were constructed using PCR amplification; the RNA libraries were quantified by qPCR, according to the qPCR Quantification Protocol Guide (San Diego, CA, USA), and were verified using the Agilent Bioanalyzer 2100.

To analyze gene expression levels and to identify alternative spliced transcripts, the RNA-Seq reads were mapped to the *Sus scrofa* genome by using TopHat [27], which can report split-read alignments across splice junctions, and gene expression was determined using the Cufflinks software [28] using default settings. The reference genome sequence of *Sus scrofa (ssc_ref_Sscrofa10*.*2)* and annotation data were obtained from the NCBI website. The transcript counts at the isoform level were calculated, and relative transcript abundances were measured in units of FPKM (fragments per kilobase of exon per million fragments mapped) by using Cufflinks. In addition, novel transcript and novel alternative splicing transcripts were identified in each sample by using the Cufflinks reference annotation based transcript assembly (RABT) method, which allows for the discovery of reference transcripts and novel transcripts on using the -g option.

### Statistical analysis

#### Experimental reproducibility

The RNA sequencing was performed only once. Additionally, 3 biological and 4 technological replicates were performed for RT-PCR, real-time PCR, and immunohistochemistry analyses. The real time RT-PCR data were analyzed by one-way analysis of variance using SPSS statistical package ver. 21.0 for Windows. A t-test was performed for comparisons between control and experimental groups. All data are expressed as the mean ± standard deviation. The null hypothesis was rejected when the probability was *P* < 0.05.

#### mRNA expression

Raw data were calculated as Fragments Per Kilobase of exon model per Millon mapped fragments (FPKM) for each transcript in each sample by Cufflinks software. We excluded transcripts where zeroed FPKM values existed for more than one sample. We added one to the FPKM value of the filtered transcript to facilitate log10 transformation. Filtered data was transformed by logarithm and normalized by the quantile normalization method. For each transcript, we calculated the fold change between case and control and determined the significant genes using a filter of |fold change| ≥4.

#### Hierarchical clustering

Hierarchical clustering analysis was also performed using complete linkage and Euclidean distance as a measure of similarity to display the expression patterns of differentially expressed genes (DEGs), which were satisfied with a filter of |fold change|≥4.

#### Multidimensional scaling

We used the multidimensional scaling (MDS) method to visualize the similarities among group pairs. MDS converts the structure in the similarity matrix to a simple geometrical picture displayed as a scatter plot. The larger the dissimilarity between two samples, the further apart the points representing the experiments will be in the plot. We applied to the Euclidean distance as the measure of the dissimilarity.

### Immunohistochemistry

For immunohistochemistry analysis, 5-day old pTestes were fixed in Bouin’s solution overnight at 4°C. Samples were subsequently washed in 70% to 100% (v/v) ethanol, embedded in paraffin, sliced into 5 μm-thick sections and mounted onto glass slides. The mounted pTestis tissue was rehydrated using xylene and 100% to 50% ethanol. To determine the expression of matrix metallopeptidase 9 (MMP9), insulin-like growth factor binding protein 3 (IGFBP3), CD14 and CD209 in the testis cells, antigen was retrieved by boiling for 30 min in tris-ethylenediamine tetra acetic acid (EDTA) solution. The antigen-retrieved tissues were stained with a 1:100 dilution of MMP9 (SC-10737; Santa Cruz Biotechnology), CD14 (Lab made, rabbit polyclonal) and CD209 (Lab made, rabbit polyclonal) antibodies followed by goat anti-rabbit IgG-HRP (SC-2004; Santa Cruz Biotechnology) and with a 1:100 dilution of IGFBP3 antibody (SC-6004; Santa Cruz Biotechnology) followed by donkey anti-goat IgG-HRP (SC-2020; Santa Cruz Biotechnology). The peroxidase substrate detection kit (SK-4100; Vector Laboratories) was used for detection of the putative pSSC marker genes according to the manufacturer’s instruction. Mouse anti-human PGP9.5 IgG (7863–1004; AbD Serotec) was used for co-immunostaining new putative porcine SSC markers with PGP9.5. Alexa Fluor^®^568 donkey anti-rabbit IgG (A-11042; Life Technologies, Carlsbad, CA, USA), Alexa Fluor^®^568 rabbit anti-goat IgG (A-11079; Life Technologies), and Alexa Fluor^®^488 goat anti-mouse IgG (A-11001; Life Technologies) were used for detecting CD14, CD209, IGFBP3, and PGP9.5. To detect nuclei, the porcine testes were treated with 4′,6-diamidino-2-phenylindol (DAPI; D9542; Sigma-Aldrich, St Louis, MO, USA) and mounting solution (S3025; DAKO) was used to fix the porcine testes. The immunostained tissues were observed under a confocal microscope (LSM 700; Carl Zeiss).

### Polyclonal antibody production

CD14 and CD209 primary antibodies were synthesized from AbFrontier (Seoul, South Korea). Peptides were designed with “NH_2_-GNPYMDPEALQHQEDPMAS-COOH” amino acid sequence near the C-terminal region and “NH_2_-DPKEPEEKTWTGPVLVERC-COOH” near the N-terminal region of CD14 and CD209, respectively. The immunization, determination of titer, and purification methods are described in supplementary information.

## Result and Discussion

### Sampling of pSSCs

**Fig 1:**
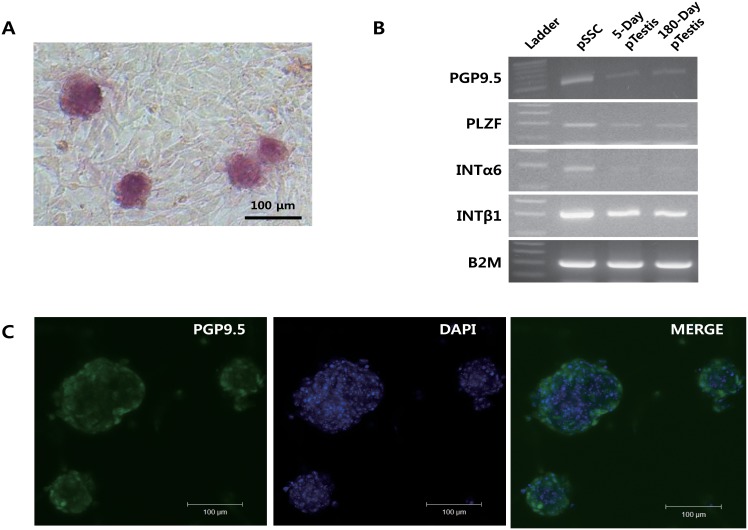
Characterization of porcine spermatogonial stem cells (pSSCs). (A) Alkaline phosphatase (AP) staining of pSSC colonies. (B) Reverse transcription-polymerase chain reaction (RT-PCR) analysis of pSSCs with undifferentiated SSC markers (PGP9.5, PLZF, integrin α6, and integrin β1). (C) Immunocytochemistry of pSSC colonies with PGP9.5 antibody.

The goal of this study was to identify the biomarkers of porcine spermatogonial stem cell that can be used to understand the biological and physiological study of spermatogenesis and early differentiation of spermatogonia. Based on our previous study [16], highly purified populations of pig spermatogonial stem cells were successfully isolated and their gene expression profiles were analyzed. Alkaline phosphatase (AP) staining of established pSSCs colonies in 31°C culture conditions showed the strong reactivity (Fig 1A), and the colonies strongly expressed PGP9.5 (Fig 1C). In addition, pSSCs colonies showed stronger expression of PGP9.5, PLZF, α6, and β1 integrins than 5- and 180-day-old pTestes (Fig 1B). Based on data presented in [16] and the current standard of determination of porcine spermatogonia, transcriptome analysis of pSSCs, 5-, and 180-day-old pTestes was performed.

### RNA sequencing of samples from pSSC, 5-, and 180-day old porcine testes

**Fig 2:**
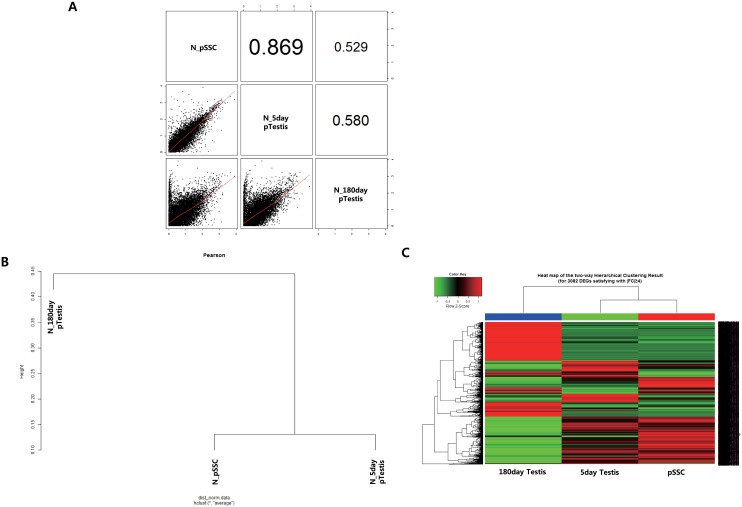
Analysis of homogeneity between porcine spermatogonial stem cells (pSSCs), 5-day porcine testis (5-day pTestis), and 180-day old porcine testis (180-day pTestis). (A) Pair-wise scatter plots of three different samples. (B) Hierarchical clustering of the three different samples. (C) Heat map representation of three different samples.

To identify the similarity of gene expression among the mRNAs from pSSCs, 5-, and 180-day-old pTestes, pairwise scatter plots were utilized. The correlation value was 0.0869 between pSSCs and 5-day-old pTestis and 0.580 between 5-day and 180-day-old pTestis (Fig 2A). The hierarchical clustering result showed that pSSCs were grouped with 5-day-old pTestis (Fig 2B and 2C).

### Differentially expressed genes in pSSC, 5-, and 180-day old porcine testes

**Fig 3:**
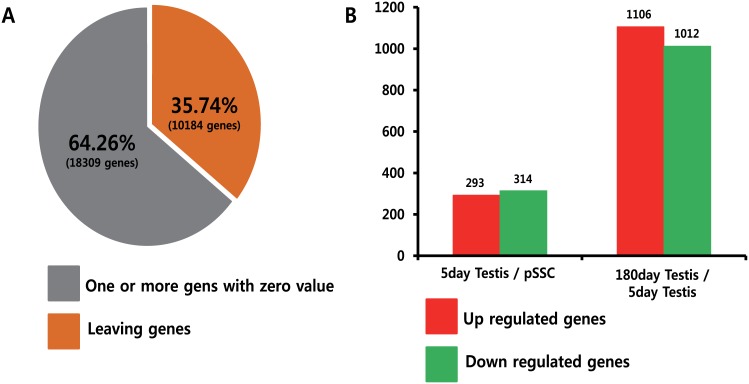
Quantitative analysis of differentially regulated genes in porcine spermatogonial stem cells (pSSCs), 5-day porcine testis (5-day pTestis), and 180-day old pTestis. (A) Pie chart representation of the percentage of genes that are significantly upregulated and downregulated in three different samples. (B) Bar chart representation of the number of genes that are significantly upregulated and downregulated in three different samples.

In the first step of the principal gene analysis, 18309 (of the total 28493) transcripts with zero FPKMs were excluded (Fig 3A). Of the remaining 10184 transcripts, 607 and 2118 transcripts had a ≥4 fold difference in gene expression in the pSSCs compared to cells from 5- and 180-day-old pTestes, respectively (Fig 3B). Among the differentially expressed genes, 293 transcripts were upregulated and 314 transcripts were downregulated in 5-day-old pTestis cells compared with pSSCs, whereas 1106 transcripts were upregulated and 1012 were downregulated in 180-day-old pTestis cells compared with 5-day-old pTestis cells (Fig 3B).

The 20 most upregulated and downregulated genes from the comparison of pSSCs to 5-day-old pTestes (Table 1) and 5 to 180-day-old pTestes (Table 2) are presented (excluding uncharacterized genes). Additional upregulated (≥4 fold) and downregulated (<4 fold) genes in each comparison analysis are listed in the supplementary information.

**Table 1:** Twenty genes (with the highest fold change) that were differentially expressed in pSSCs compared with 5-day old pTestis cells.

temp_ID	transcript_ID	gene	description	5-day testis/pSSC.fc	chromosome
**Upregulated genes**					
rna22416	NM_001038004.1	MMP9	matrix metallopeptidase 9 (gelatinase B, 92-kDa gelatinase, 92kDa type IV collagenase)	-202.5144304	17
rna19024	XM_003132811.1	ADAMDEC1	ADAM-like, decysin 1	-69.63632317	14
rna13659	NM_214023.1	SPP1	secreted phosphoprotein 1	-62.81446742	8
rna575	XM_001927036.4	COL12A1	collagen, type XII, alpha 1	-54.55227654	1
rna19807	NM_213945.1	PLAU	plasminogen activator, urokinase	-46.66264521	14
rna23036	NM_001005156.1	IGFBP3	insulin-like growth factor binding protein 3	-37.90158818	18
rna3669	NM_001129972.1	CD209	CD209 molecule	-36.20854348	2
rna14251	NM_001166229.1	MMP1	matrix metallopeptidase 1 (interstitial collagenase)	-30.14299096	9
rna5867	NM_001244261.1	EFEMP1	EGF containing fibulin-like extracellular matrix protein 1	-27.98453879	3
rna978	NM_001244536.1	THBS1	thrombospondin 1	-23.43092947	1
rna17690	NM_214201.1	GPX1	glutathione peroxidase 1	-21.88974227	13
rna13493	XM_003129219.3	QRFPR	pyroglutamylated RFamide peptide receptor	-21.4555113	8
rna3556	NM_001105288.1	COL5A3	collagen, type V, alpha 3	-16.714075	2
rna20213	XM_003359366.1	DUSP5	dual specificity phosphatase 5	-16.43734793	14
rna4729	NM_001146300.1	FSCN1	fascin homolog 1, actin-bundling protein (Strongylocentrotus purpuratus)	-15.54146804	3
rna15855	NM_001206347.1	POSTN	periostin, osteoblast specific factor	-15.29643787	11
rna21319	NM_001244315.1	ARL4C	ADP-ribosylation factor-like 4C	-14.57090368	15
rna1588	NM_001163998.1	ANXA1	annexin A1	-13.35714703	1
rna13674	XM_003357094.2	PTPN13	protein tyrosine phosphatase, non-receptor type 13 (APO-1/CD95 (Fas)-associated phosphatase)	-13.05835055	8
rna4401	NM_001097445.2	CD14	CD14 molecule	-12.19022716	2
**Downregulated genes**					
rna20175	NM_214428.1	CYP17A1	cytochrome P450 17A1	1532.243521	14
rna13940	NM_001144841.1	HBB	hemoglobin, beta	759.0978348	9
rna3250	NM_213970.1	INSL3	insulin-like 3 (Leydig cell)	695.4371183	2
rna779	NM_214430.1	CYP19A2	cytochrome P450 19A2	251.8932554	1
rna12267	NM_214019.1	DHRS4	dehydrogenase/reductase (SDR family) member 4	189.2986973	7
rna20654	NM_213755.2	STAR	steroidogenic acute regulatory protein	146.4769738	15
rna12495	NM_001243402.1	RDH12	retinol dehydrogenase 12 (all-trans/9-cis/11-cis)	143.016292	7
rna9200	NM_213913.1	HSD11B2	hydroxysteroid (11-beta) dehydrogenase 2	118.6958888	6
rna8171	NM_001163997.1	MYL6	myosin, light chain 6, alkali, smooth muscle and non-muscle	80.42465149	5
rna20177	XM_003359357.1	AS3MT	arsenic (+3 oxidation state) methyltransferase	79.03076896	14
rna1070	NM_001001770.1	CYB5A	cytochrome b5 type A (microsomal)	77.66790365	1
rna15415	NM_213912.3	TNNI1	troponin I type 1 (skeletal, slow)	70.92907077	10
rna7040	XM_003481433.1	RHBG	Rh family, B glycoprotein	60.80151791	4
rna5909	NM_214449.1	LHCGR	luteinizing hormone/choriogonadotropin receptor	57.91914584	3
rna13446	NM_001195363.1	OSAP	ovary-specific acidic protein	50.96013903	8
rna15700	NM_001123075.1	AKR1C4	aldo-keto reductase family 1, member C4 (chlordecone reductase; 3-alpha hydroxysteroid dehydrogenase, type I; dihydrodiol dehydrogenase 4)	46.58927222	10
rna15708	NM_001038626.2	AKR1CL1	aldo-keto reductase family 1, member C-like 1	42.73718267	10
rna2403	XM_003122495.2	CTSF	cathepsin F	42.6957037	2
rna14623	NM_214064.1	FMO1	flavin containing monooxygenase 1	34.69624949	9
rna16743	XM_003131573.3	ACSF2	acyl-CoA synthetase family member 2	32.68981351	12

**Table 2:** Twenty genes (with the highest fold change) that were differentially expressed in 180-day old pTestis cells compared with 5-day old pTestis cells

temp_ID	transcript_ID	gene	description	180-day testis/5-day testis.fc	chromosome
**Upregulated genes**					
rna7335	XM_003125791.1	ANKRD35	ankyrin repeat domain 35	1042.173275	4
rna5156	XM_003354608.2	TNP2	transition protein 2 (during histone to protamine replacement)	983.2982125	3
rna5151	NM_214253.1	PRM1	protamine 1	946.1636991	3
rna6526	NM_214255.1	ODFP	outer dense fiber protein	726.3327634	4
rna5152	NM_214252.1	PRM2	protamine 2	692.2115135	3
rna17774	XM_001928213.2	PHF7	PHD finger protein 7	548.4183607	13
rna17686	XM_003132204.3	CCDC36	coiled-coil domain containing 36	513.8496863	13
rna7172	NM_001008685.1	SMCP	sperm mitochondria-associated cysteine-rich protein	443.5745283	4
rna11941	NM_213782.1	PGK2	phosphoglycerate kinase 2	318.6499228	7
rna7450	NM_001243436.1	ADORA3	adenosine A3 receptor	316.2668766	4
rna6711	NM_001206401.1	DNAJC5B	DnaJ (Hsp40) homolog, subfamily C, member 5 beta	303.3817502	4
rna14602	NM_001177920.1	SPATA19	spermatogenesis associated 19	302.8779925	9
rna18503	NM_001172369.1	ROPN1	rhophilin associated tail protein 1	266.5005732	13
rna390	NM_001190247.1	SPACA1	sperm acrosome associated 1	196.3337759	1
rna20971	NM_001197305.1	STAT4	signal transducer and activator of transcription 4	184.1411917	15
rna12646	NM_001177908.1	SPATA7	spermatogenesis associated 7	173.5459516	7
rna15632	NM_001159312.1	SPAG6	sperm associated antigen 6	168.3108318	10
rna8578	XM_003126533.1	ACRBP	acrosin binding protein	161.3776945	5
rna19001	NM_001162888.1	PEBP4	phosphatidylethanolamine-binding protein 4	160.4191096	14
rna7658	XM_003125964.3	PKDREJ	polycystic kidney disease (polycystin) and REJ homolog (sperm receptor for egg jelly homolog, sea urchin)	135.1556887	5
**Downregulated genes**					
rna13940	NM_001144841.1	HBB	hemoglobin, beta	-128.9317969	9
rna9200	NM_213913.1	HSD11B2	hydroxysteroid (11-beta) dehydrogenase 2	-90.5267327	6
rna15415	NM_213912.3	TNNI1	troponin I type 1 (skeletal, slow)	-62.14388074	10
rna5909	NM_214449.1	LHCGR	luteinizing hormone/choriogonadotropin receptor	-61.11530707	3
rna20175	NM_214428.1	CYP17A1	cytochrome P450 17A1	-52.06466615	14
rna20177	XM_003359357.1	AS3MT	arsenic (+3 oxidation state) methyltransferase	-42.66665102	14
rna8613	NM_214088.1	CCND2	cyclin D2	-41.7317786	5
rna7040	XM_003481433.1	RHBG	Rh family, B glycoprotein	-39.52662828	4
rna618	NM_001243029.1	LIPG	lipase, endothelial	-38.54923815	1
rna20941	NM_001243297.1	COL3A1	collagen, type III, alpha 1	-36.56016994	15
rna10838	NM_001243354.1	DHCR24	24-dehydrocholesterol reductase	-36.54408882	6
rna12267	NM_214019.1	DHRS4	dehydrogenase/reductase (SDR family) member 4	-35.80189866	7
rna1070	NM_001001770.1	CYB5A	cytochrome b5 type A (microsomal)	-35.51291887	1
rna19177	NM_213967.1	SCARB1	scavenger receptor class B, member 1	-35.26115186	14
rna3638	NM_001038644.1	ANGPTL4	angiopoietin-like 4	-30.56363482	2
rna2967	NM_214170.1	CD59	CD59 molecule, complement regulatory protein	-26.60766432	2
rna16920	NM_214214.1	CCL2	chemokine (C-C motif) ligand 2	-26.37682455	12
rna20654	NM_213755.2	STAR	steroidogenic acute regulatory protein	-26.23993747	15
rna779	NM_214430.1	CYP19A2	cytochrome P450 19A2	-25.49701562	1
rna16540	NM_001243807.1	FKBP10	FK506 binding protein 10, 65 kDa	-25.38872465	12

Interestingly, most of the upregulated genes in 180-day-old pTestes compared to 5-day-old pTestes are related to spermatogenic differentiation, including spermatocytogenesis and spermiogenesis of germ cells. For examples, protamine 1 and 2 are expressed only during the post meiotic stages [29] and bind to the phosphate backbone of DNA, and the ratio of these proteins during spermatogenesis is important to determine the shape of the sperm head. An imbalance of this ratio causes subfertility or infertility [30]. Transition protein 2 (TNP2) is a key molecule during histone to protamine replacement. Defects of TNP2 in testes showed an abnormal development of the sperm head with acrosomal defects, impaired migration of the spermatozoa in the female genital tract, and defects of normal fertilization by loss of ability to penetrate the zona pellucida [31, 32]. Spermatogenic germ cell development is a predominant activity rather than testes growth in 180-day-old pTestes, therefore increased expression of these genes is predictable, suggesting that the transcriptome analysis in this study to identify differential gene expression is valid for identifying new putative gene expression markers in pSSCs.

### Expression of putative SSC markers

**Fig 4:**
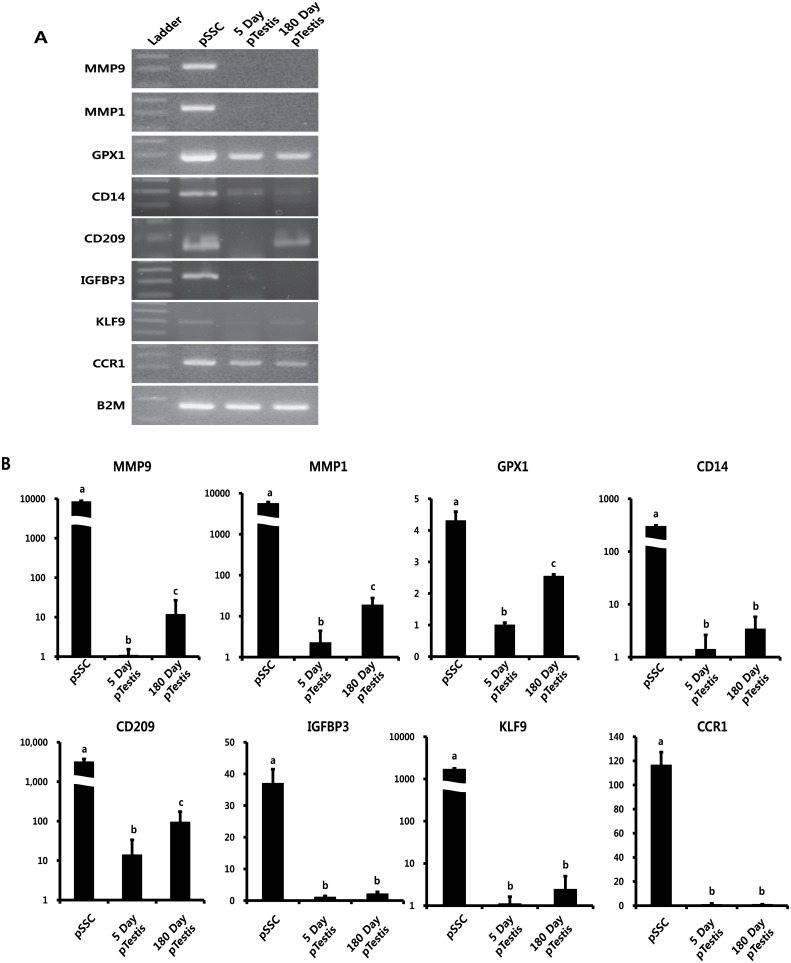
Expression of porcine spermatogonial stem cell-specific marker genes. (A) RT-PCR analysis of putative spermatogonial stem cell markers. (B) Real time RT-PCR analysis of putative spermatogonial stem cell markers.

**Fig 5:**
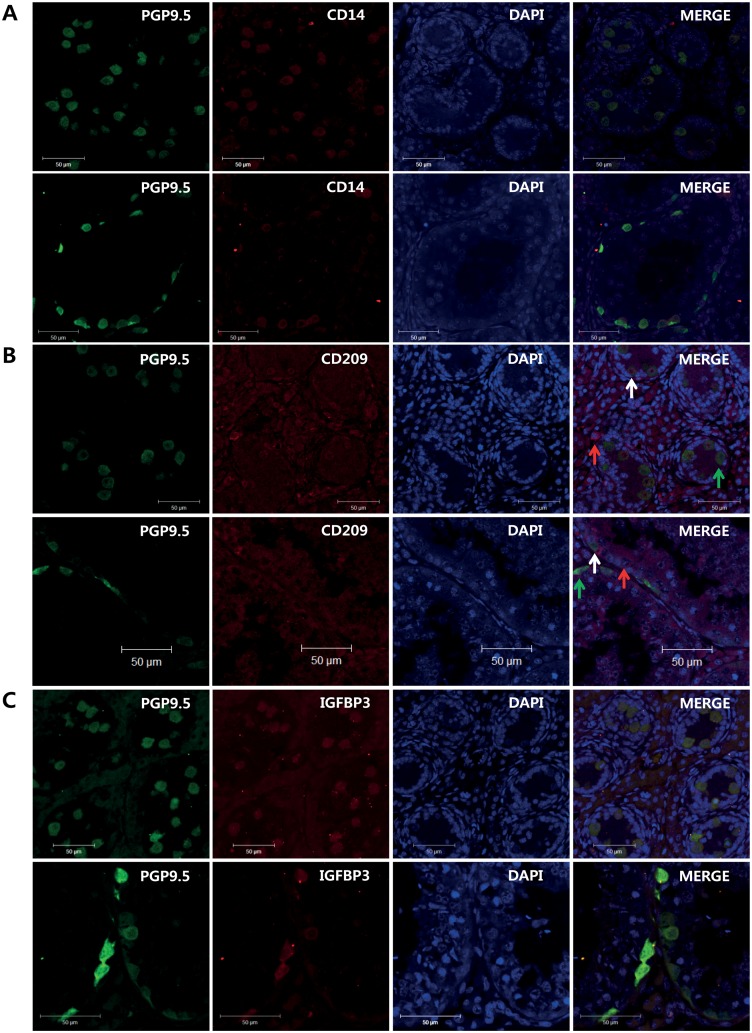
Expression of porcine spermatogonial stem cell specific markers. Immunohistochemistry analysis of CD14, CD209, and IGFBP3 protein expression in 5- and 180-day old pTestes. (A) CD14 expression in 5- (upper) and 180- (lower) day-old pTestes. (B) CD209 expression in 5- (upper) and 180- (lower) day-old pTestes. (C) IGFBP3 expression in 5- (upper) and 180- (lower) day-old pTestes. Red and green arrows indicate cells that only expressed CD209 or PGP9.5, respectively. White arrows indicate cells that expressed both CD209 and PGP9.5.

Among the 20 highly expressed genes in pSSCs, the expression patterns and levels of MMP1, MMP9, GPX1, CD14, CD209, IGFBP3, KLF9, and CCR1 were evaluated by RT-PCR and real time RT-PCR in pSSC, 5-, and 180-day-old pTestes. The band intensities of these genes were greater in pSSCs than in 5- and 180-day-old pTestes (Fig 4A). In addition, real time RT-PCR confirmed the higher relative expression level of these genes in pSSCs than in 5- and 180-day-old pTestes (Fig 4B). To identify the expression and localization of these proteins in 5- and 180-day-old pTestis tissues, immunohistochemistry with antibodies for CD14, CD209, and IGFBP3 antibodies was performed. Due to the lack of species-specific compatibility of commercial antibodies for MMP1, MMP9, GPX1, KLF9, and CCR1 to porcine, only CD14, CD209, and IGFBP3 were tested. CD14, CD209, and IGFBP3 were expressed specifically in spermatogonia (Fig 5). However, the CD209 antibody also stained cells in the interstitial area. In porcine testes, three types of germ cells were detected in the basement membrane: cells that stained for both PGP9.5 and CD209 (white arrow), cells that stained for only PGP9.5 (green arrow), and cells that expressed only CD209 (red arrow) (Fig 5B). In addition, expression of MMP9 in 5-day old testes was also determined (S1 Fig). Together with RT-PCR and real time RT-PCR data, immunohistochemistry clearly confirmed the increased expression of these genes in spermatogonia in 5- and 180-day-old pTestes, suggesting that these genes can be used as putative biomarkers of the early stage of porcine spermatogonia.

Interestingly MMPs are among the highly expressed genes in pSSCs. MMPs are a family of enzymes playing an essential role in proteolytic degradation of the extracellular matrix and are involved in matrix remodeling in relation to embryonic developmental processes, inflammation and tumor invasion and metastasis [33–36]. MMP2 and MMP9 are known to bind to heparan sulfate proteoglycans, and CCR1, one of the highly expressed genes in pSSCs. CCR1^+^ myeloid cells appear to enhance tumor invasion by producing metalloproteinases, MMP9 and MMP2 [37]. Both MMP9 and MMP2 are positioned to interact with the cell surface adhesion molecules or receptors and to regulate the turnover of these molecules [38]. MMP9 is abundantly expressed in both Sertoli cells and gonocytes and MMP1 is localized to Sertoli cells in human fetal testis [39]. However, in the present study, MMP9 expression was observed specifically in spermatogonia of 5-day-old pTestes and in *in vitro* cultured pSSCs. In rodent testes, colocalization of MMP2, laminin γ3, and β1 integrin was observed in the basal ectoplasmic region and activated when the spermatid detached and migrated from the epithelium [40, 41]. Interestingly, treatment with specific MMP2 and MMP9 inhibitors delayed detachment of spermatid by inhibition of proteolysis of laminin [40]. Proteolysis of laminin through MMPs may be essential in migration of meiotic germ cells. In rodent testes, proximal extracellular matrix (ECM), which is mainly composed of type IV collagen, laminins, heparin sulfate proteoglycan and entactin, constituted the basement membrane of seminiferous tubule, and contact with Sertoli cells and spermatogonia [42]. Taken together, high MMP expression levels in both *in vitro* cultured pSSCs and spermatogonia in 5-day-old pTestes may play a role in the migration of spermatogonia by degradation of the surrounded ECM, because, *in vivo*, gonocytes and spermatogonia are initially located at a distance from the basement membrane of spermatic cord and seminiferous tubule at early ages, but they move to settle on the basement membrane before puberty.

In addition, MMP1 can degrade insulin like growth factor binding proteins (IGFBPs) into fragments with low affinity for insulin like growth factor (IGF), thus the bioactivity of IGFs to cell surface IGF receptors [43]. Although the precise role of IGF axis and their regulatory mechanism in germ cell development, expressions of IGFs have been reported in human Sertoli cells and primary spermatocytes [44] and equine testes [45]. In addition, spermatogonia can be an important source of testicular IGFs that may regulate Sertoli cells through a paracrine factor [46], and spermatogonia proliferation and differentiation as an autocrine factor [47].

In addition, urokinase plasminogen activator (u-PA) inhibited the activities of IGFBP3 by proteolysis and increased the bioactivities of IGFs [48, 49]. Therefore expression of MMPs, u-PA, and IGFBP3 in pSSCs may be relevant to regulation of IGFs function in the early stages of spermatogenesis.

CD14, a 55-kDa glycoprotein found on the cell surface of myeloid cells [50], is also known as a receptor for lipopolysaccharides (LPS) or a complex of LPS and LPS binding protein [51–53] and regulates LPS-induced cell activation [54]. *CD14* mRNA was upregulated in male reproductive organs, including testis, epididymis, and seminal vesicles by LPS-treatment [55]. Although the role and mechanism of CD14 in testes are not identified yet, flow cytometric analyses confirmed the expression of CD14 on a subpopulation of cryptorchidism testis cells in enriched SSC [56]. In addition, the antioxidant enzymes, especially glutathione peroxidase-1 (GPX1), were upregulated in *in vitro* cultured pSSCs. Additionally, GPX1 was also upregulated in type A spermatogonia in rodents [57].

CD209 is a type II membrane protein with a C-type lectin extracellular domain [58]. CD209 plays an important role in establishing the initial contact between dendritic cells and the resting T lymphocytes through its recognition of intercellular adhesion molecule-3. Immunofluorescence analysis revealed that CD209 is only expressed on a small percentage of CD14 positive blood cells, whereas it is highly expressed on immature dendritic cells in peripheral tissues and *in vitro* derived monocyte-derived dendritic cells [58, 59]. The staining patterns were almost identical to those observed in the present study (Fig 5B).

In conclusion, transcriptome analysis of *in vitro* cultured pSSCs, 5-, and 180-day-old pTestis cells were conducted to discover putative biomarkers for porcine spermatogonia and spermatogonia stem cells. Among the highly expressed genes in pSSCs compared to 5-day-old pTestis cells, the expression ofMMP9, MMP1, GPX1, CCR1, IGFBP3, CD14, CD209, and KLF9 was evaluated by RT-PCR and real time RT-PCR to confirm that expression of these genes was specific to pSSCs. In addition, localization and expression of CD14, CD209, and IGFBP3 were determined by immunohistochemistry in spermatogonia of 5- and 180-day-old pTestes, suggesting that newly identified putative biomarkers for porcine spermatogonia can be useful to study early events of spermatogenesis and to enrich spermatogonia *in vitro*.

## Supporting Information

S1 Fig Expression of porcine spermatogonial stem cell specific markers. (DOCX)

S1 File Genes that were differentially expressed in pSSC, 5- and 180-day old porcine testis cells. (ZIP)

S1 Table Primers used for the reverse transcription-polymerase chain reaction (RT-PCR) of cDNA from pSSC, 5- and 180- day old porcine testis cells. (DOCX)

S1 Text Polyclonal Antibody Production for CD14 and CD209. (DOCX)
