# Supplementary material for: Identification of Putative Biomarkers for the Early Stage of Porcine Spermatogonial Stem Cells Using Next-Generation Sequencing
Source: PLoS One. 2016 Jan 22;11(1):e0147298. doi: 10.1371/journal.pone.0147298 (PMC4723225; doi:10.1371/journal.pone.0147298)

**S1 Fig. Expression of porcine spermatogonial stem cell specific markers.** Immunohistochemistry analysis of MMP9, IGFBP3, CD14, and CD209 protein expression in 5-day old porcine testes. The arrows indicate porcine spermatogonial stem cells.


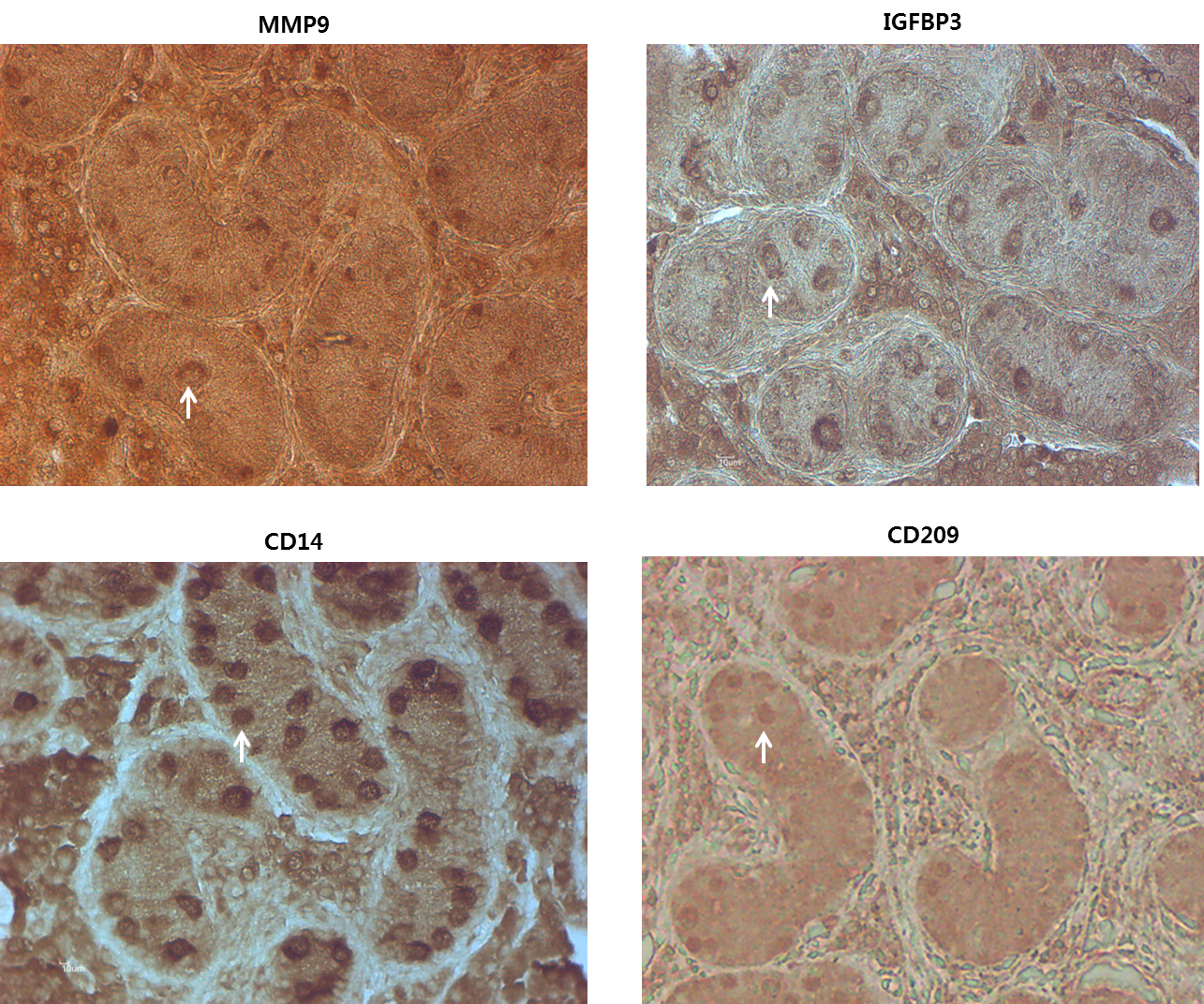

Supplement: S1 Fig — (DOCX) [file pone.0147298.s001.docx]
