# Supplementary material for: Identification of Putative Biomarkers for the Early Stage of Porcine Spermatogonial Stem Cells Using Next-Generation Sequencing
Source: PLoS One. 2016 Jan 22;11(1):e0147298. doi: 10.1371/journal.pone.0147298 (PMC4723225; doi:10.1371/journal.pone.0147298)
Supplement: S1 Table — (DOCX) [file pone.0147298.s003.docx]

**S1 Table. Primers used for the reverse transcription-polymerase chain reaction (RT-PCR) of cDNA from pSSC, 5- and 180- day old porcine testis cells.**

| **Gene** | **Forward Primer** | **Reverse primer** |
| --- | --- | --- |
| PGP9.5 | 5'-GAGATGCTGAACAAAGTGCTG-3' | 5'-CATGGTTCACCGGAAAAGG-3' |
| PLZF | 5'-GGCTCGGTATCTCAAGAACATC-3' | 5'-ACTGCCCTATGGTCATCAA ACT-3' |
| INTα6 | 5'-TCATGGATCTGCAAATGGAA-3' | 5'-TAATAACAGGCCGGGATCTG-3' |
| INTβ1 | 5'-GGGGATGAGGTTCAGTTTGA-3' | 5'-CACACTCAAACGTCCCATTG-3' |
| MMP9 | 5'-CTGCAATGTGGACATCTTCG-3' | 5'-ACTTGGCGTCCAGAGAAGAA-3' |
| MMP1 | 5'-TGTTCTCACTCCAGGGAACC-3' | 5'-TTCCTCCAGGTCCATCAAAG-3' |
| GPX1 | 5'-CAAGAATGGGGAGATCCTGA-3' | 5'-GATAAACTTGGGGTCGGTCA-3' |
| CCR1 | 5'-CTGGACCTGGCCATACAAGT-3' | 5'-AGAAGCCAGCAGAGAGTTCG-3' |
| IGFBP3 | 5'-gctgaaccacctcaagttcc-3' | 5'-acttatccacgcaccagcag-3' |
| CD14 | 5'-ACCACCCTCAGACTCCGTAAT-3' | 5'-ATAGGTCCAGGGTGGTGAGAG-3' |
| CD209 | 5'-ATTGGGTTTCTTCATGCTCCT-3' | 5'-CATTCATCCAGGTCATCTGCT-3' |
| KLF9 | 5'-CAGTGTCTGGTTTCCATTTCG-3' | 5'-GTCGGTACTTGTTCAGGTCCA-3' |
| B2M | 5’-TTCACACCGCTCCAGTAG-3’ | 5’-CCAGATACATAGCAGTTCAGG-3’ |
