# Supplementary material for: Identification of Putative Biomarkers for the Early Stage of Porcine Spermatogonial Stem Cells Using Next-Generation Sequencing
Source: PLoS One. 2016 Jan 22;11(1):e0147298. doi: 10.1371/journal.pone.0147298 (PMC4723225; doi:10.1371/journal.pone.0147298)
Supplement: S1 Text — (DOCX) [file pone.0147298.s004.docx]

**S1 Text. Polyclonal Antibody Production for CD14 and CD209**

CD14 and CD209 primary antibodies were synthesized from AbFrontier (Seoul, South Korea). Peptides were designed with the amino acid sequence “NH_2_-GNPYMDPEALQHQEDPMAS-COOH” near the C-terminal region and “NH_2_-DPKEPEEKTWTGPVLVERC-COOH” near the N-terminal region of CD14 and CD209, respectively (Fig. 1).

Fig. 1. Amino acid sequences of CD14 and CD209. (A) CD14 sequence. (B) CD209 sequence. Boxed regions are epitopes for antibody production.

**
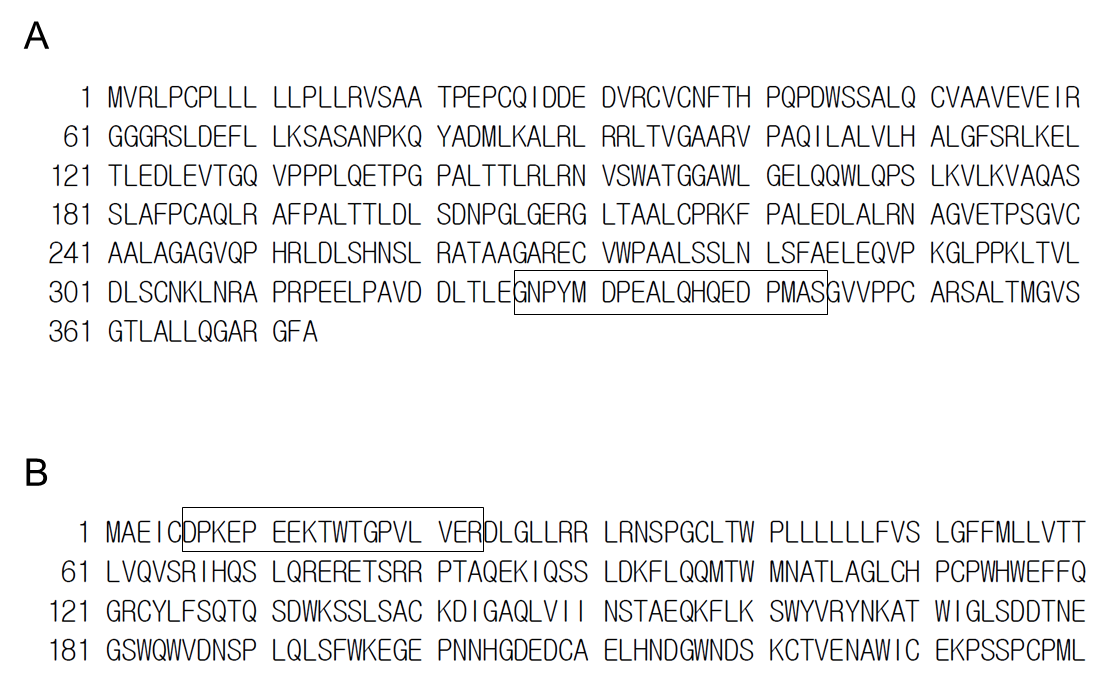
**

Immunization was performed as shown in Table 1. The complete Freund’s adjuvant (CFA, Sigma Aldrich, F5881) and incomplete Freund’s adjuvant (IFA, Sigma Aldrich, F5506) were injected with CD14 and CD209 antigen peptides subcutaneously (SC) as indicated in Table1.

Table1. Immunization protocol

| **Time** | **Description** | **Note** |
| --- | --- | --- |
| Week 0 (pre-immune bleeding ) | Pre-immune serum collection (ear vein) | ~about 1 mL serum/Rabbit |
| Week 0 (pre-immunization) | 1 mg/Rabbit in Complete Freund’s Adjuvant (CFA) (SC injection) |  |
| Week 4 (1^st^ immunization) | 500 μg/Rabbit in Incomplete Freund’s Adjuvant (IFA) (SC injection) |  |
| Week 5 (1^st^ bleed) | 1^st^ production bleed (ear vein) 1^st^ ELISA test | about 1 mL serum/Rabbit |
| Week 6 (2^nd^ immunization) | 500 μg/Rabbit in Incomplete Freund’s Adjuvant (IFA) (SC injection) |  |
| Week 7 (2^nd^ bleed) | 2^nd^ production bleed (ear vein) 2^nd^ ELISA test | about 1 mL serum/Rabbit |
| Week 8 (3^rd^ immunization) | 500 μg/Rabbit in Incomplete Freund’s Adjuvant (IFA) (SC injection) |  |
| Week 9 (final bleed) | Euthanasia (Heart puncture), Final ELISA test | about 50 mL serum/Rabbit |

The antibody titer was determined 3 times using enzyme-linked immunosorbent assay (ELISA) as shown in Table 2 and the titer data of serially diluted antibodies are presented in Fig. 2.

Table 2. ELISA protocol

|  | **Description** |
| --- | --- |
| 1 | Dilute the antigen to 2 μg/mL with coating buffer and aliquot 50μL in individual wells (96-well plate) and incubate for overnight at 4℃. |
| 2 | Discard the coating solution. |
| 3 | Add 250 μL 2% BSA/PBS and block at 37℃for 1 h. |
| 4 | Wash once with TBS-T and add 100 μL primary antibody (serum) into individual wells and incubate for 2 h at 37℃. |
| 5 | Wash 3 times with TBS-T and dilute the secondary antibody (goat anti-rabbit IgG(Fc) HRP) at a 1:5,000 ratio. Add 50 μL into individual wells and incubate for 1 h at 37℃. |
| 6 | Wash 5 times with TBS-T and add 50 μL TMB (color reagent) in each well. When the color changes, add 100 μL stop solution (1N H_2_SO_4_) and measure the O.D. value at 450 nm. |
| Reagent | Coating buffer (500 mL): 0.2 M Na_2_CO_3_ 80 mL, 0.2 M NaHCO_3_170 mL, D.W up to 500 mL  1X TBS-T (pH7.4): 10 mM Tris, 0.15 M NaCl, 0.1% Tween20  Blocking Solution: 2% bovine serum albumin (BSA) in PBS |

Fig. 2. ELISA results of CD14 and CD209 antibodies.


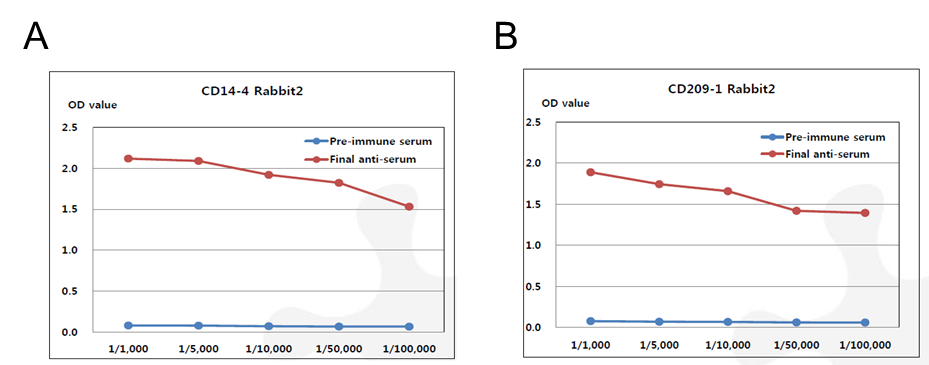


The rabbit IgG was then purified using the rapid antibody purification kit (CELL BIOLABS, INC., AKR-160) as shown in Table 3. The concentration of the purified antibodies was measured using the Bradford assay and final stocks at a concentration of 10 mg/mL were stored at -20℃ in TBS-T containing 0.02% sodium azide.

Table 3. Antibody purification protocol

|  | **Description** |
| --- | --- |
| 1 | Invert the Protein A Agarose Column several times to resuspend the agarose beads. Remove the top cap from the Protein A column first, then remove the bottom cap to allow the storage solution to drain through the column. |
| 2 | Slowly add 5 mL of Binding Buffer to the top of the resin. Allow the column to drain until liquid level drops to the resin level. |
| 3 | Apply up to 5 mL of diluted antibody sample to the equilibrated Protein A column and allow the antibody sample to flow through the column. |
| 4 | Wash column with 10 mL of Binding Buffer and allow it to drain through the column. |
| 5 | Elute the bound antibody with 5 mL of Elution Buffer and collect 1 mL elution fractions. |
| 6 | Immediately add 50 μL of Neutralization Buffer to each of the 1 mL elution fractions. |
